# Cannabis for the Treatment of Inflammatory Bowel Disease: A True Medicine or a False Promise?

**DOI:** 10.5041/RMMJ.10390

**Published:** 2020-01-30

**Authors:** Timna Naftali, Michael Dor

**Affiliations:** 1 Institute of Gastroenterology and Hepatology, Meir Hospital Sapir Medical Center, Kfar Saba, Israel; 2 The Sackler School of Medicine, Tel Aviv University, Tel Aviv, Israel; 3 Former Senior Medical Cannabis Consultant of the Medical Cannabis Unit, Israeli Ministry of Health, Jerusalem, Israel

**Keywords:** Cannabis, Crohn’s disease, IBD, ulcerative colitis

## Abstract

Cannabis is the most widely used recreational drug worldwide and is used by some patients with inflammatory bowel disease (IBD) to ameliorate their disease. Whereas epidemiological studies indicate that as many as 15% of IBD patients use cannabis, studies inspecting cannabis use in IBD are few and small. We have conducted several studies looking at the use of cannabis in IBD. In Crohn’s disease, we demonstrated that cannabis reduces the Crohn’s disease activity index (CDAI) by >100 points (on a scale of 0–450).Two small studies in ulcerative colitis showed a marginal benefit. However, no improvement was observed in inflammatory markers or in endoscopic score in either disease. Many questions regarding cannabis use in IBD remain unanswered. For example, cannabis is a complex plant containing many ingredients, and the synergism or antagonism between them likely plays a role in the relative efficacy of various cannabis strains. The optimal doses and mode of consumption are not determined, and the most common form of consumption, i.e. smoking, is unacceptable for delivering medical treatment. Cannabis is a psychotropic drug, and the consequences of long-term use are unknown. Despite all these limitations, public opinion regards cannabis as a harmless drug with substantial medical efficacy. In Israel, the number of licenses issued for the medical use of cannabis is rising rapidly, as are the acknowledged indications for such use, but good-quality evidence for the effectiveness of cannabis is still lacking. Further studies investigating the medical use of cannabis are urgently needed.

## BACKGROUND

Cannabis is the most widely used recreational drug worldwide. The cannabis plant contains as many as 100 phytocannabinoids, as well as other ingredients such as terpenes and flavonoids.^1^ The phytocannabinoids exert their effect through the endocannabinoid system (ECS), which is an endogenous system with an important role in modulating mood, memory, reward homeostasis, immune regulation, and energy balance.^2^ The best-known phytocannabinoids are Δ9-tetrahydrocannabinol (THC), responsible for the psychotropic effect of cannabis, and cannabidiol (CBD), which does not have a central effect but was shown to have an anti-inflammatory effect.^3^

## REPORTS OF CANNABIS USE IN INFLAMMATORY BOWEL DISEASE

Many animal and laboratory studies demonstrated that cannabis can ameliorate inflammation in inflammatory bowel disease (IBD).^4^ Consequently, there are many epidemiological studies and anecdotal reports about cannabis use in IBD patients. Various studies demonstrated that the prevalence of cannabis use among IBD patients varies between 12% and 15%, although a much higher percentage of patients (50%–60%) report ever using cannabis during their lifetime.^5^,^6^ Patients claim that cannabis ameliorates their symptoms, including improvement in diarrhea, abdominal pain, and appetite^7^; however, most studies contain no information about the dose and mode of cannabis consumption. We conducted an observational study of 127 IBD patients who were using cannabis by license from the Ministry of Health in Israel and found that most patients were satisfied with a monthly dose of 30 g and that 70% were consuming cannabis by smoking it, whereas the others were consuming it orally, mostly in the form of oil.^8^ Nevertheless, since patients are using many different varieties of cannabis, with different content of cannabinoids, obtaining more accurate information is difficult.

## RANDOMIZED CONTROLLED TRIALS OF CANNABIS IN IBD

In view of the many reports about cannabis use in IBD, it is surprising that very few randomized controlled trials (RCTs) have been conducted. Two Cochrane reviews found only three trials performed in Crohn’s disease^9^ and only two in ulcerative colitis.^10^ This can be partly explained by the fact that investigating cannabis use is inherently difficult. The large variations between different cannabis strains and the many different modes of cannabis consumption make properly standardized cannabis treatment hard to achieve.

In the first RCT, 21 Crohn’s disease patients were randomized to receive either cannabis flowers or a placebo containing 23% THC. A clinical response, defined as a decrease in the Crohn’s disease activity index (CDAI) by >100 points (on a scale of 0–450) was observed in 10/11 (91%) subjects in the cannabis group and 4/10 (40%) in the placebo group (*P*=0.028).^11^ Another trial looking at the use of CBD for Crohn’s disease found no significant difference in the CDAI between the study and the placebo groups (220±122 and 216±121, respectively, *P*=NS).^12^

The first RCT to report cannabis use in ulcerative colitis included 60 patients who received a CBD-rich cannabis botanical extract for 10 weeks. Remission rates were similar for the CBD (28%) and placebo (26%) groups. Although CBD is usually well tolerated, in this study side effects led to a 40% protocol deviation in the study group.^13^ We performed a study of cannabis in ulcerative colitis at the Meir Medical Center, demonstrating that the disease activity index (Lichtiger score) after 8 weeks of cannabis treatment was 4 in cannabis participants compared with 8 in the placebo group (*P* between groups 0.001).^10^

There are no studies regarding the maintenance of remission with cannabis in either Crohn’s disease or ulcerative colitis.

The Israeli Gastroenterological Association issued recommendations for the use of cannabis in IBD. These were adopted by the Israeli Ministry of Health. These recommendations state that since the evidence of cannabis efficacy in IBD is still lacking, cannabis should be used only as a compassionate treatment in patients for whom the established forms of treatment have failed—that is, patients who still suffer from the active disease despite treatment by biologics, and who are not candidates for surgery.

## REGULATION OF MEDICAL CANNABIS IN ISRAEL

Despite the lack of scientifically sound evidence, cannabis use is rapidly gaining popularity and legitimacy throughout the world. Medical cannabis treatment was introduced in Israel in 1994, but until 2001 it was approved for only 64 patients. During the last decade, pressure from the media and politicians, together with increasing awareness of physicians and patients, pushed the numbers up. Consequently, the number of permits increased from 12,000 in 2013 to 60,000 in 2019. New instructions published by the Ministry of Health allowed each specialist to recommend the treatment within the limits of his/her specialization. The recommendations were examined by qualified physicians in the Ministry, and 90% of the requests were granted. A license was sent to the patient specifying the dispensary allocated to them, the amount of cannabis, and the consumption method approved. The dispensary supplied cannabis to the patients and instructed them on how to use it. However, there was no specification of the strain of cannabis to be used or the content of THC allowed. Consequently, the treating physician ended up prescribing a treatment but having no control of the doses of the psychoactive substance the patient would consume. During those years more than 80,000 licenses were issued, and, at the time of writing, more than 50,000 are active.

Regarding the various indications for cannabis use so far, 40% of the patients were oncology patients, 30% suffered from intractable pain after the failure of all conventional treatments, and 2,000 patients (4%) were treated for post-traumatic stress disorder after failure of at least 3 years of all conventional, medical, and psychological treatments. More than 1,000 patients (2%) were treated for Crohn’s disease and 150 for ulcerative colitis. More than 1,000 patients (2%) were treated for fibromyalgia (an indication that brought a lot of professional objection). The other patients suffered from neurological disorders, autoimmune diseases, and others (data from M.D., former medical adviser to the Israeli Minister of Health on Cannabis).

## THE MINISTRY OF HEALTH CANNABIS REFORM

Lately, in an attempt to define the prescription of cannabis more accurately, the Ministry of Health issued a list of allowed cannabis variations and is removing the dispensing of cannabis from cannabis producers to pharmacies. The allowed variations include flowers or oil, *Cannabis sativa* or *C. indica*, and various proportions of THC and CBD, ranging from 3% to 20%. Thus, the physician prescribing cannabis can define the exact dose of THC and CBD.

In parallel, during the last years, the Cannabis Unit of the Israeli Ministry of Health initiated a set of new regulations intended for quality control assurance. The previous system was based on a direct supply of cannabis from the grower to the patients. The new system included strict quality control, high manufacturing standards, and distribution of cannabis products through pharmacies.

A structured process of introducing new indications was initiated. The process is quite complicated, and the implementation was delayed by a court decision for several months.

In an effort to establish more scientifically sound evidence about the medical role of cannabis, a research committee, chaired by Professor Rafael Mechulam, approved more than 400 research projects, including 60 clinical studies. New research is evaluating the possible use of cannabis in the treatment of opioid addiction and the treatment of other psychiatric disorders. New indications explored in recent years include autism in children and intractable epilepsy of childhood; approximately 1,000 children in each group have been treated, with significant success. New ways of administration are being developed, starting from new inhalation devices,^14^ and continuing with topical preparations for psoriasis and atopic dermatitis.^15^,^16^ New manufacturing methods using nanotechnology are also being investigated.

## CONCLUSION

The use of medical cannabis is rapidly increasing, and physicians are faced with an increasing demand from patients to prescribe it. Sadly, this is not accompanied by scientifically sound evidence regarding the efficacy, if any, of cannabis treatment. Very little is known about the effect of cannabis, the significance of various cannabinoid combinations, or the mode of cannabis consumption. On the other hand, we cannot afford to ignore the many reports about the positive effect of cannabis. The current treatment for IBD is successful in about 60% of patients, so if indeed there is a potential for another medication this should be explored using rigorous and scientifically sound methods. Only by conducting large well-designed randomized controlled trials will we be able to benefit from the potential of this plant.

## Abbreviations

CBD: cannabidiol
CDAI: Crohn’s disease activity index
ECS: endocannabinoid system
IBD: inflammatory bowel disease
RCT(s): randomized controlled trial(s)
THC: Δ9-tetrahydrocannabinol.
